# Supplementary material for: Chemodiversity of Calophyllum inophyllum L. oil bioactive components related to their specific geographical distribution in the South Pacific region
Source: PeerJ. 2019 May 22;7:e6896. doi: 10.7717/peerj.6896 (PMC6535043; doi:10.7717/peerj.6896)
Supplement: Supplemental Information 1 — Supplementary tables (S1–S3) and figures (S1–S10). [file peerj-07-6896-s001.docx]

**SUPPORTING INFORMATION**

1. Table S1 **Voucher specimens deposited in at the South Pacific Regional Herbarium**
2. Table S2 **HPLC (1100 Agilent) analytical method and parameters used for *C. inophyllum* resin analysis**
3. Table S3 **Sample’s deposited in the NCBI Gene Bank with corresponding accession numbers and gene region**
4. Figure S1 **^1^H NMR Tamanolide E, fraction 1**
5. Figure S2 **HSQCed Tamanolide E, fraction 1**
6. Figure S3 **HMBC Tamanolide E, fraction 1**
7. Figure S4 **DEPTQ135 Tamanolide E, fraction 1**
8. Figure S5 **NOESY Tamanolide E, fraction 1**
9. Figure S6 **FTIR Tamanolide E, fraction 1**
10. Figure S7 **FTIR enlargement Tamanolide E, fraction 1**
11. Figure S8 **HR-ESI-MS Tamanolide E, fraction 1**
12. Figure S9 **^1^H NMR Tamanolide E, fraction 2**
13. Figure S10 **DEPTQ135 Tamanolide E, fraction 2**

**Table S1: Voucher specimens deposited in at the South Pacific Regional Herbarium (SPRH)**

| Sample Location | Sample Identity | SPRH voucher number |
| --- | --- | --- |
| Fiji South 1 | **F-S1** | **29401** |
| Fiji West 1 | **F-W1** | **29402** |
| Fiji East 1 | **F-E1** | **29403** |
| Fiji North 1 | **F-N1** | **29404** |
| Fiji Rotuma 1 | **F-R1** | **29405** |

Table S2: **HPLC (1100 Agilent) analytical method and parameters used for Tamanu resin analysis**

| Column | Silica (Interchim Uptishere Strategy SI 5µm 250X4.6mm) |
| --- | --- |
| Phase | Normal phase |
| Elution | gradient |
| Flow | 1mL/min |
| Method | 0 min: cyclohexane (87.5%)/ ethyl acetate (12.5%)  0 to 5 min: cyclohexane (90%)/ ethyl acetate (10%)  5 to 60 min: cyclohexane (87.5%)/ ethyl acetate (12.5%) |
| Sample Concentration | 1g/L |
| Volume of injection | 10µL |
| Run analysis | 60min |
| sample Solvent | Cyclohexane (87.5%)/ Ethyl acetate (12.5%) |
| Cleansing vial for the injection needle | Cyclohexane (87.5%)/ Ethyl acetate (12.5%) |
| Wavelength | 360nm (quantification), 400nm (reference), 100nm (Reference wavelengths)  250nm (quantification), 400nm (reference), 100nm (Reference wavelengths) |
| Peak width | >0,013min (response delay = 0.25s for 20Hz) |
| Spectra width | 190 to 400nm (2nm stepwise) |
| Acquisition | UV and visible light |

Table S3: **Sample’s deposited in the NCBI Gene Bank with corresponding accession numbers and gene region**

| Samples | Accession No. | Genus species | Chloroplast gene region |
| --- | --- | --- | --- |
| New Caledonia 5 | KT384372 | ***Calophyllum inophyllum*** | accD |
| Fiji East 1 | KT384372 | ***Calophyllum inophyllum*** | accD |
| Tahiti Hitiaa | KT384378 | ***Calophyllum inophyllum*** | accD |
| Fiji Rotuma | KT384376 | ***Calophyllum inophyllum*** | accD |
| Fiji South 5 | KT384374 | ***Calophyllum inophyllum*** | accD |
| Fiji West 1 | KT384375 | ***Calophyllum inophyllum*** | accD |
| Tahiti Patio | KT384377 | ***Calophyllum inophyllum*** | accD |
| Tahiti Avera | KT369680 | ***Calophyllum inophyllum*** | accD |
| Tahiti Avera | KT369681 | ***Calophyllum inophyllum*** | accD |
| Rotuma 2 | KT716506 | ***Calophyllum inophyllum*** | PsaA-Ycf3 spacer |
| Rotuma 4 | KT716507 | ***Calophyllum inophyllum*** | PsaA-Ycf3 spacer |
| Fiji East 1 | KT716508 | ***Calophyllum inophyllum*** | PsaA-Ycf3 spacer |
| Fiji East 2 | KT716510 | ***Calophyllum inophyllum*** | PsaA-Ycf3 spacer |
| Fiji East 5 | KT716510 | ***Calophyllum inophyllum*** | PsaA-Ycf3 spacer |
| Fiji West 2 | KT716511 | ***Calophyllum inophyllum*** | PsaA-Ycf3 spacer |


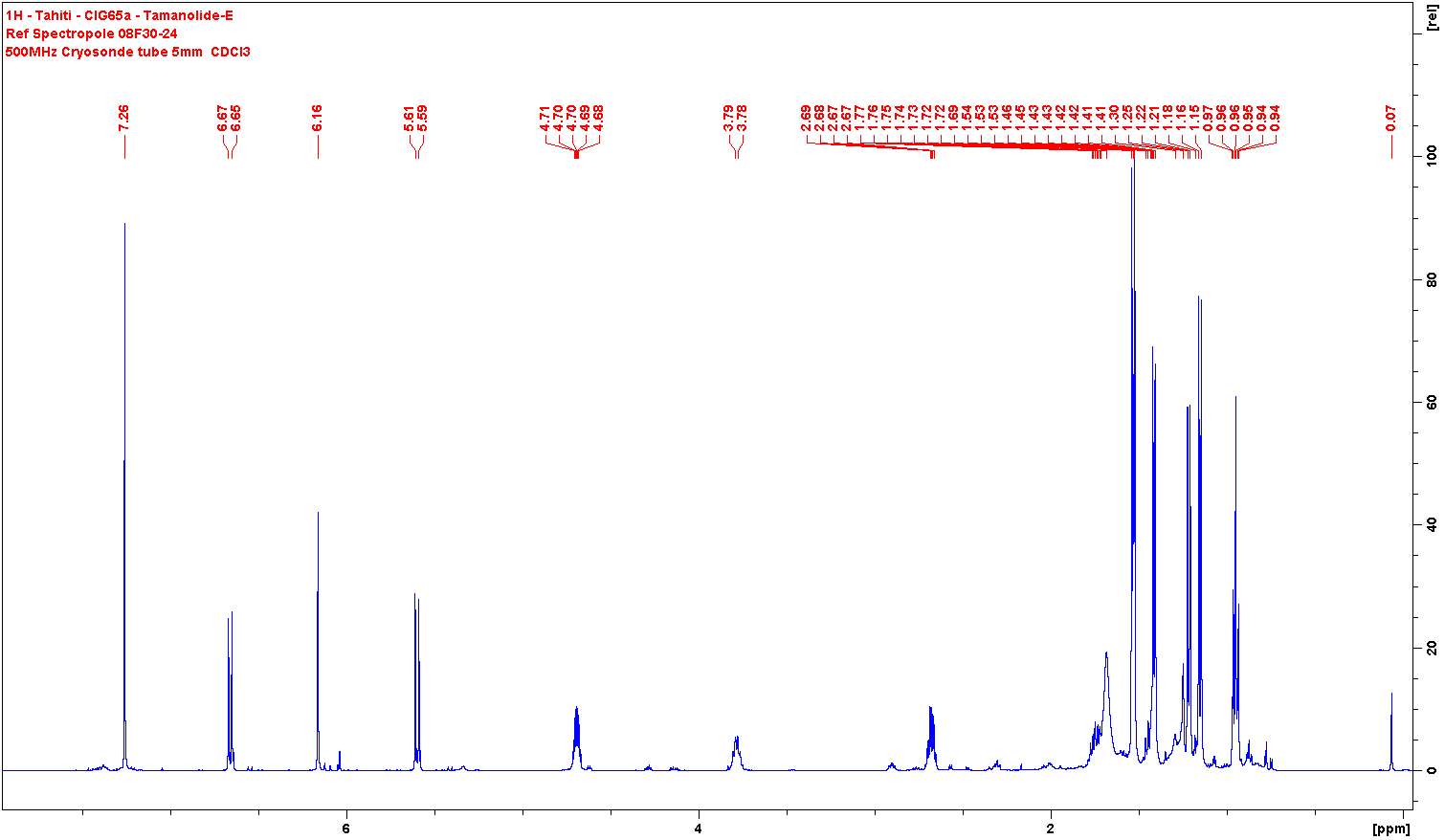


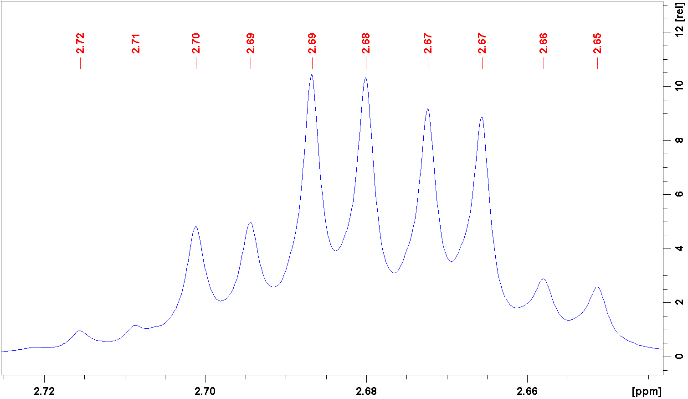

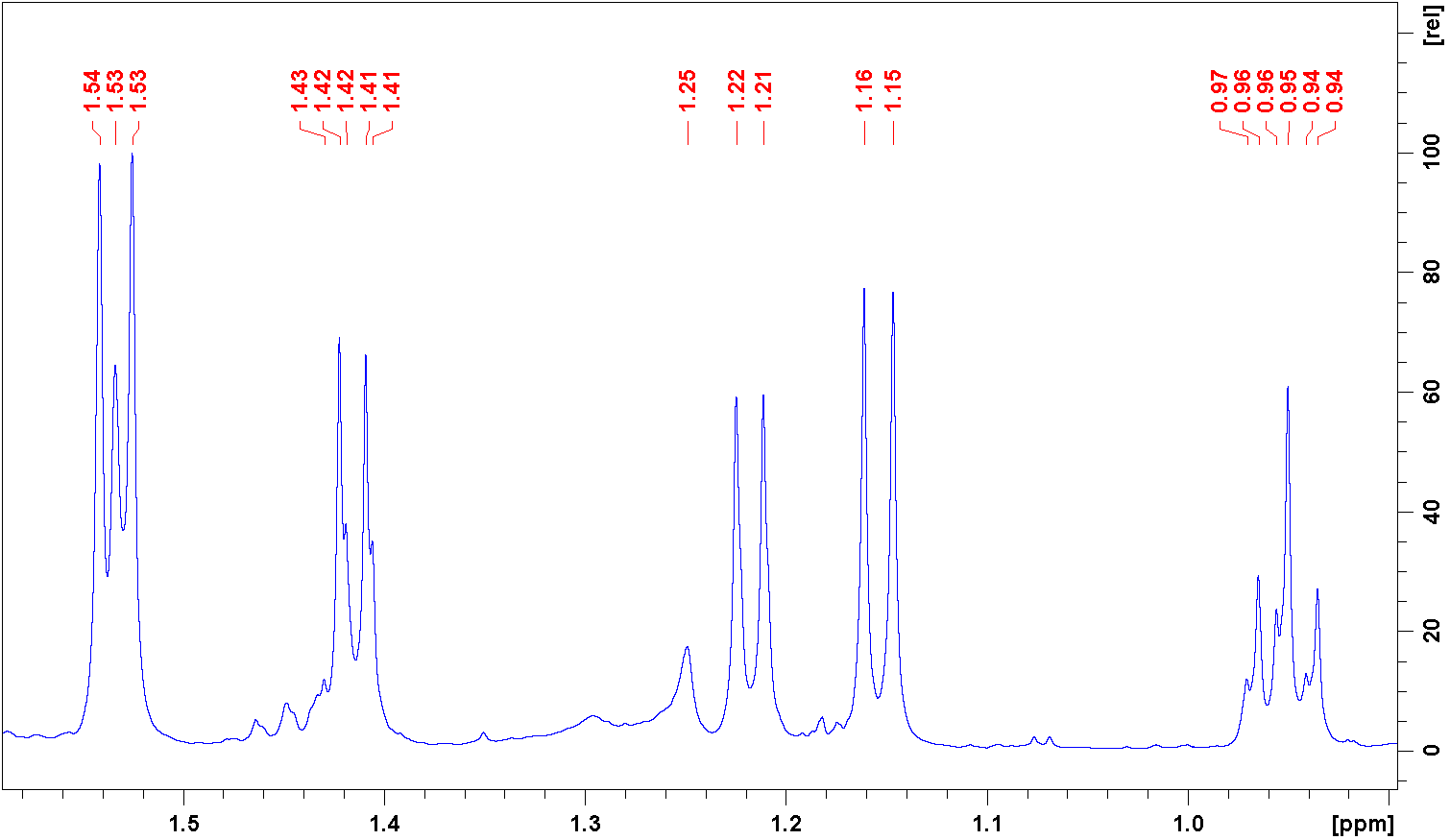


Figure S1 **^1^H NMR Tamanolide E, fraction 1**


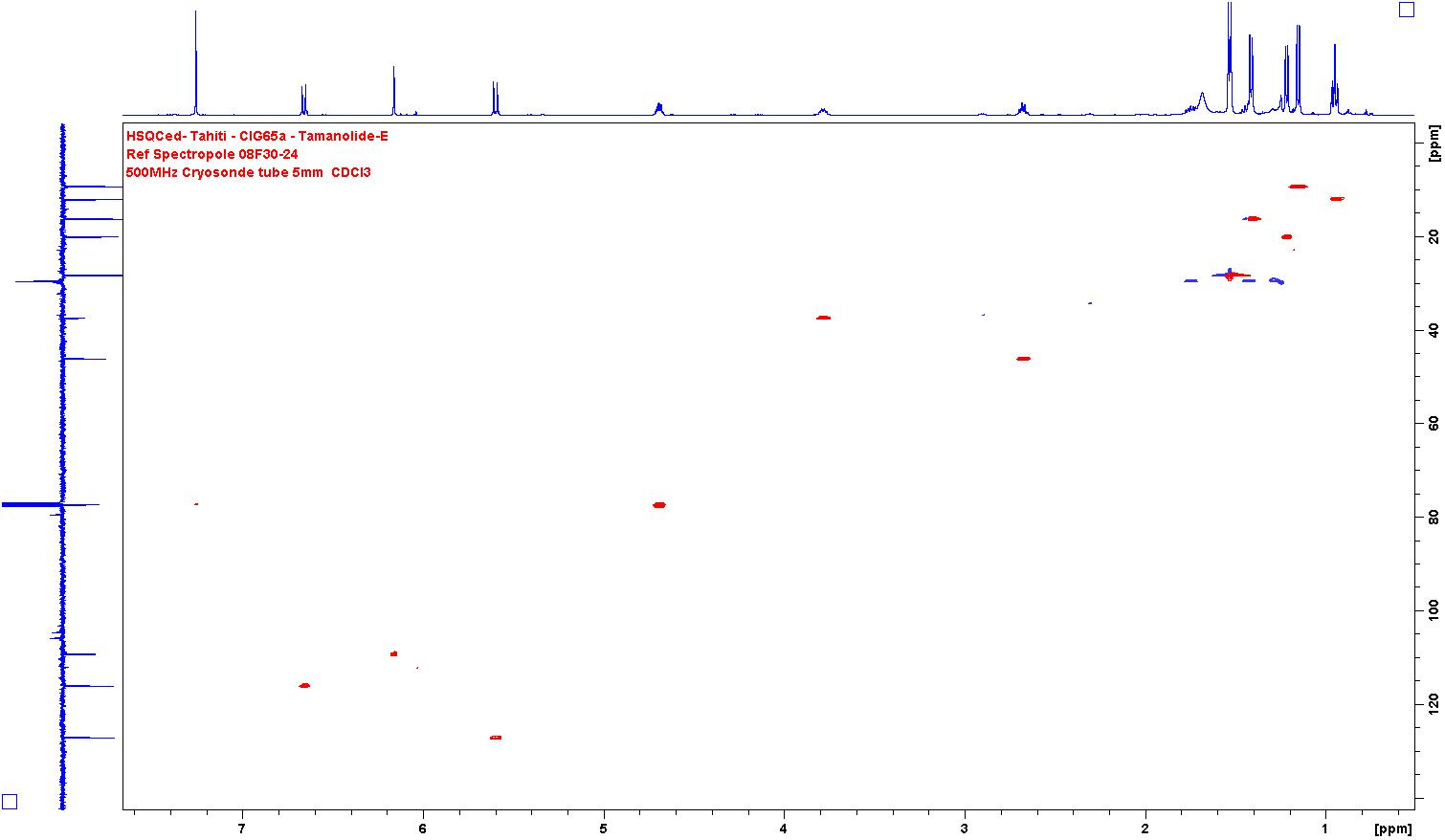


Figure S2 **HSQCed Tamanolide E, fraction 1**


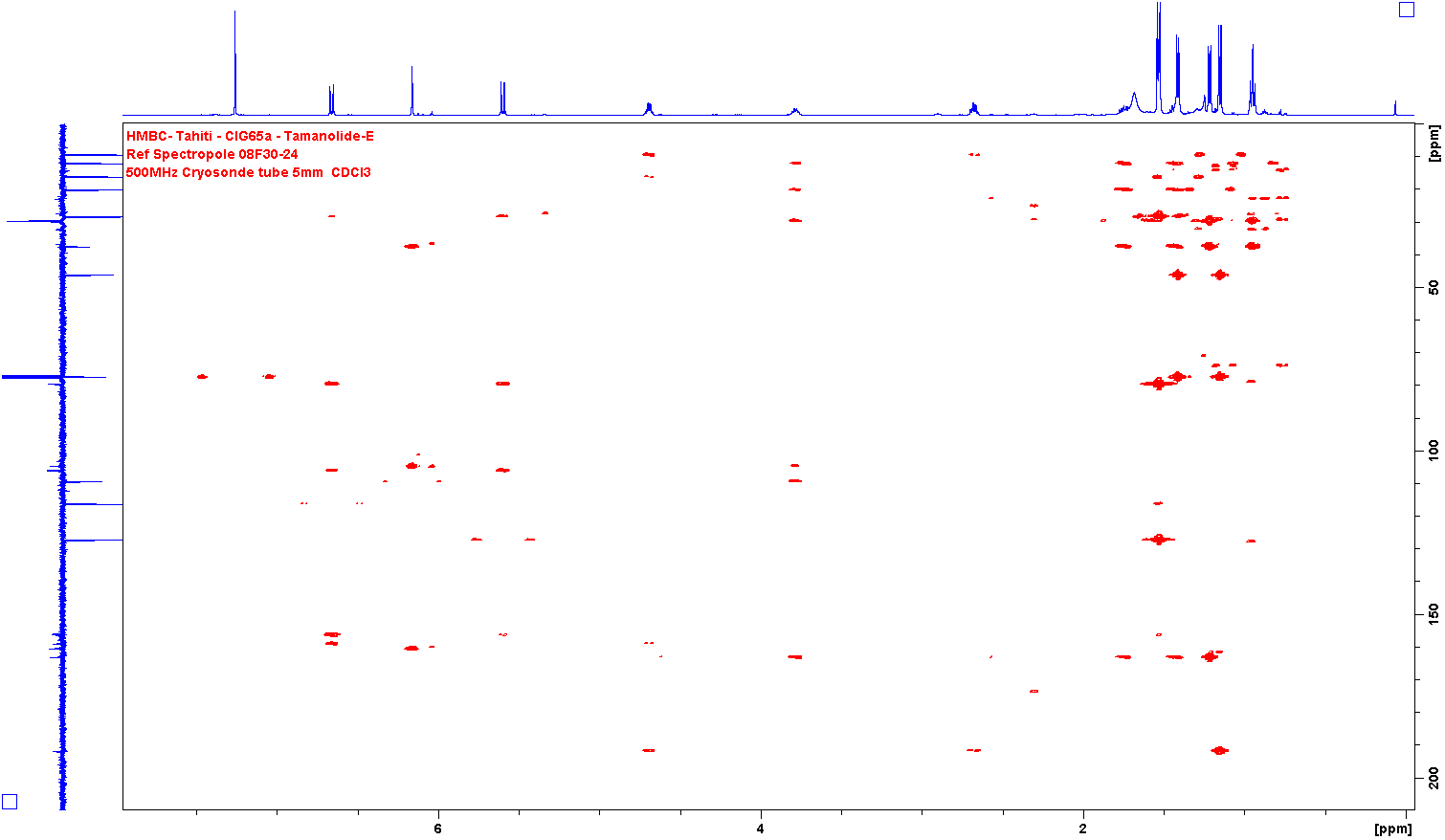


Figure S3 **HMBC Tamanolide E, fraction 1**


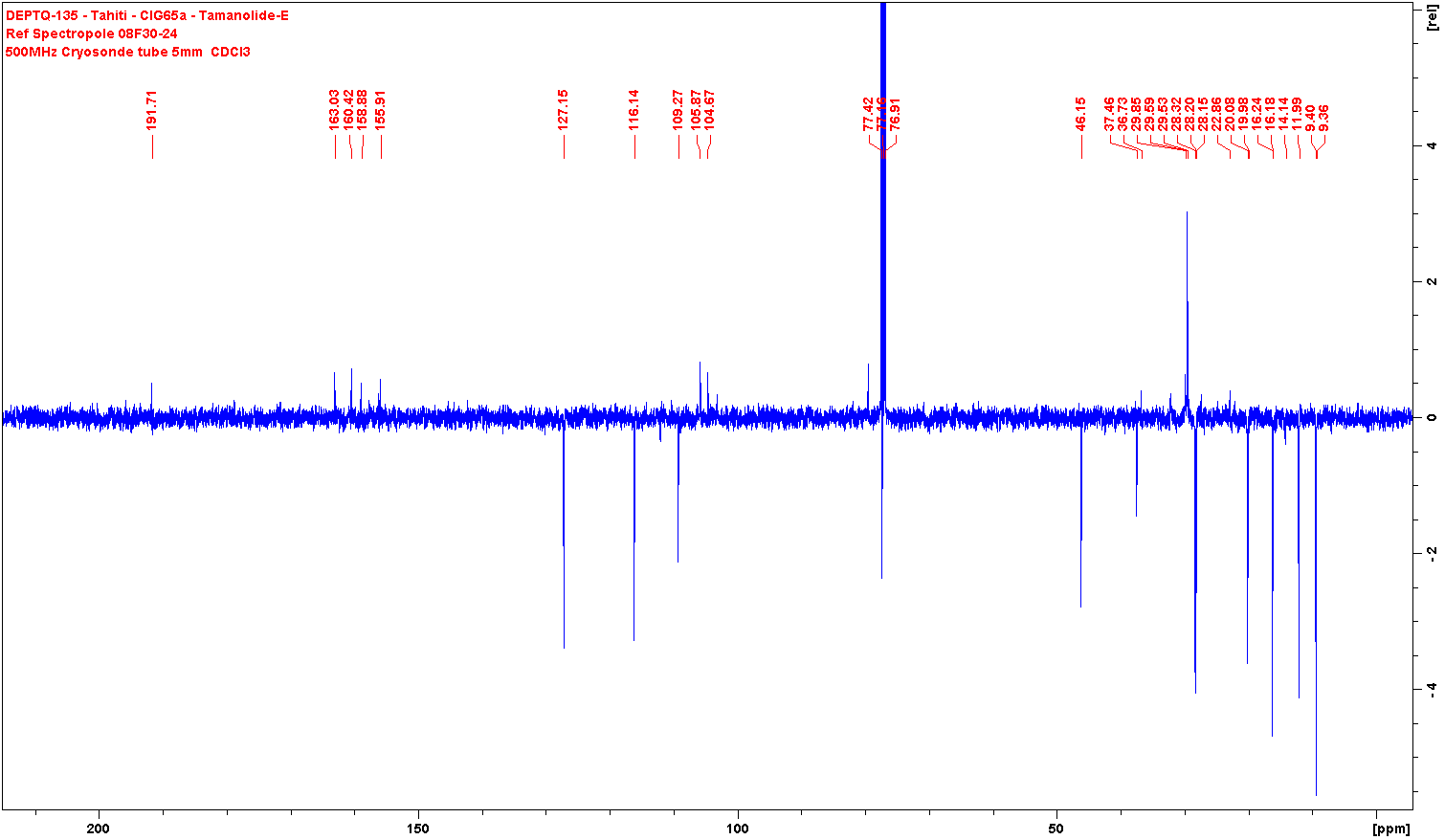


Figure S4 **DEPTQ135 Tamanolide E, fraction 1**


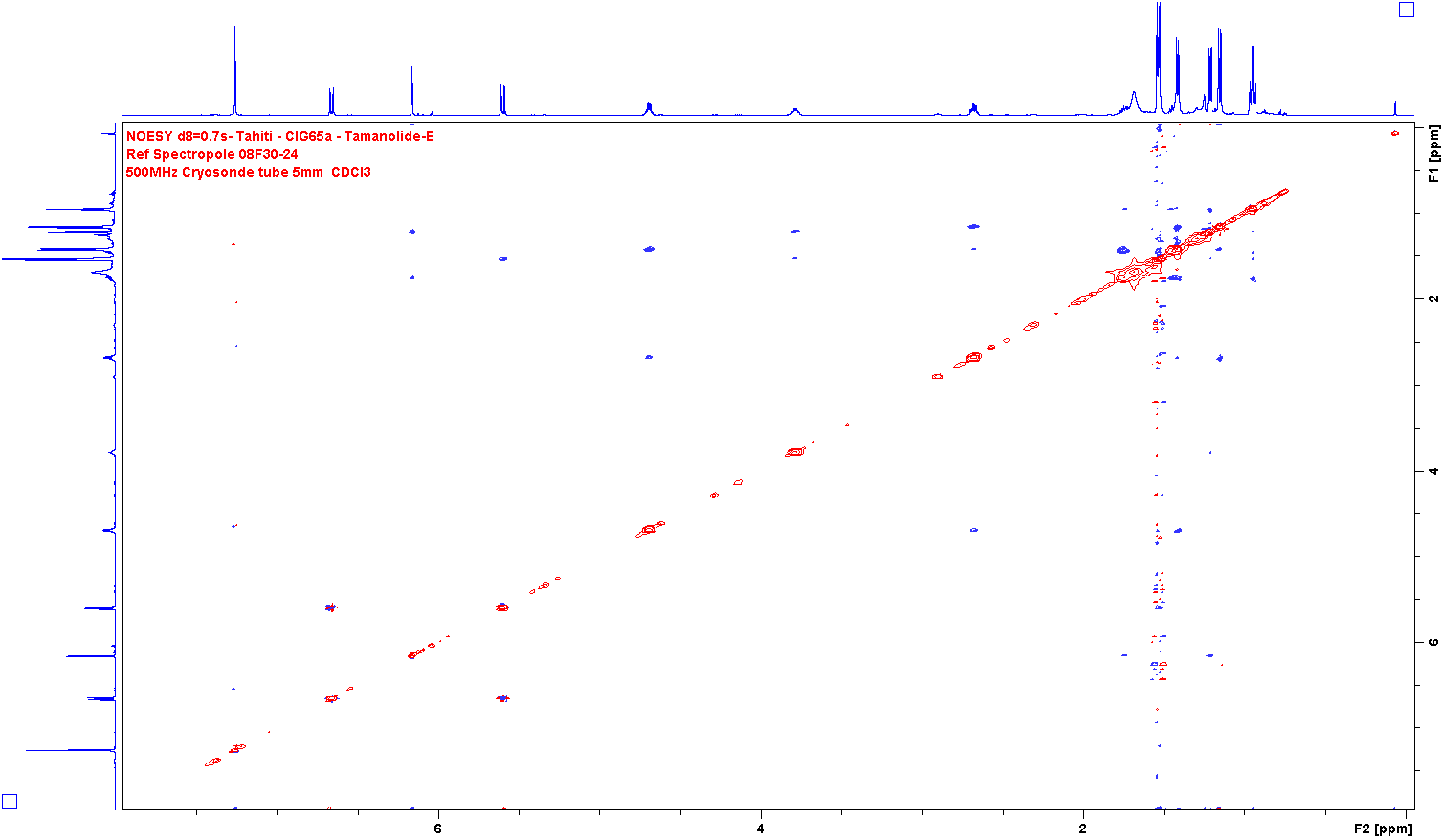


Figure S5 **NOESY Tamanolide E, fraction 1**


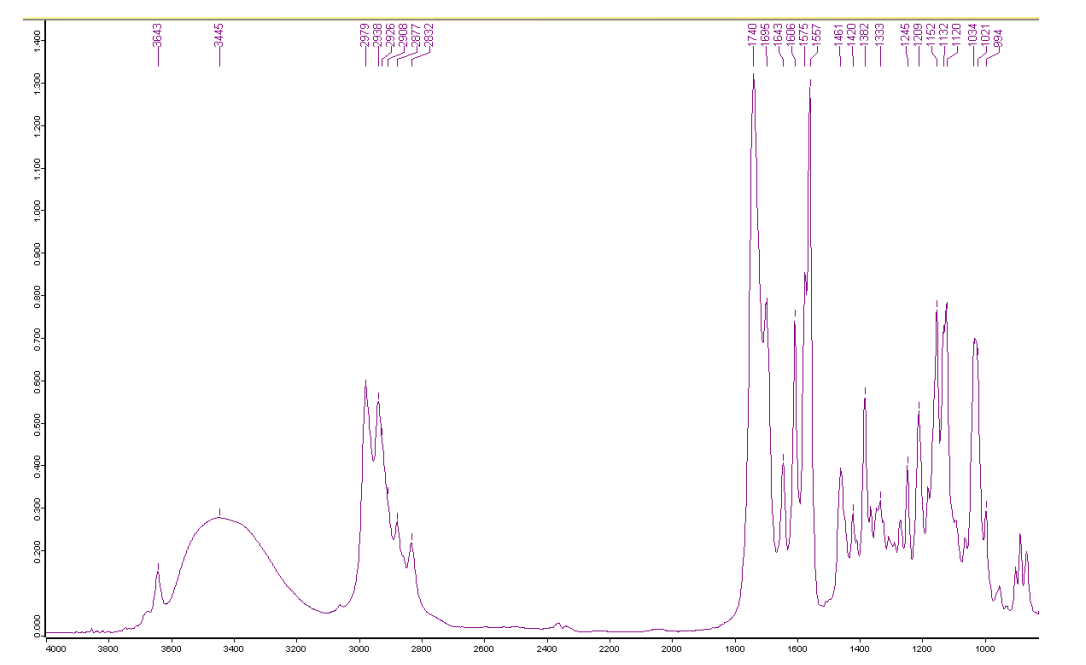


Figure S6 **FTIR Tamanolide E, fraction 1**


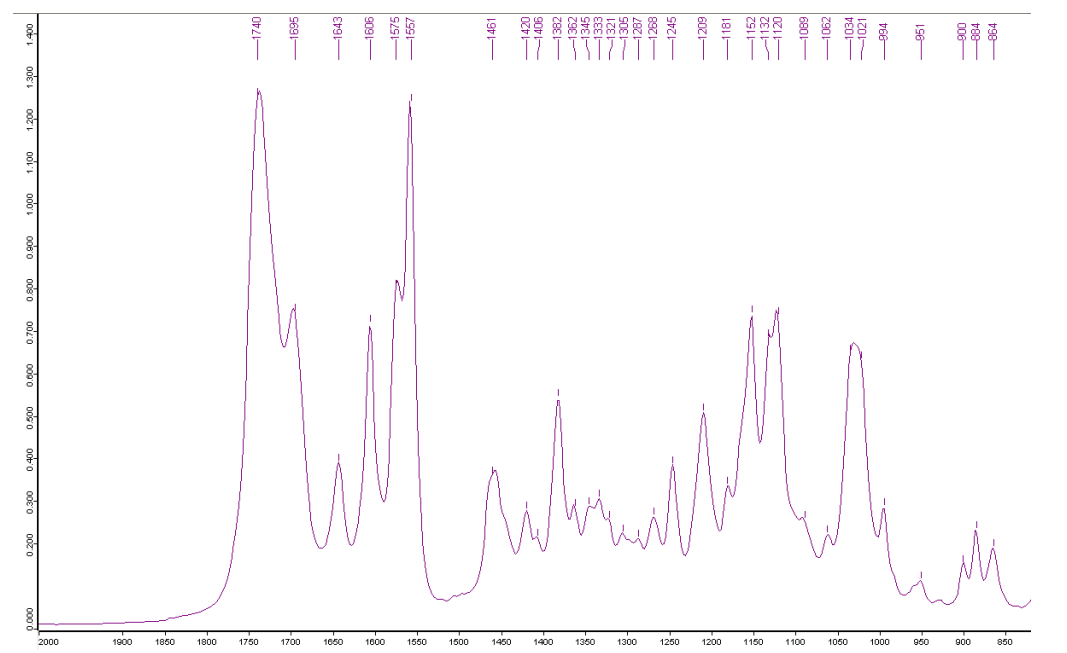


Figure S7 **FTIR enlargement Tamanolide E, fraction 1**

|  |
| --- |

Figure S8 **HR-ESI-MS Tamanolide E, fraction 1.** Detection of the targeted ion at m/z 383.1850 (pink) and control peaks (brown) m/z 344.2278 et m/z 388.2540.


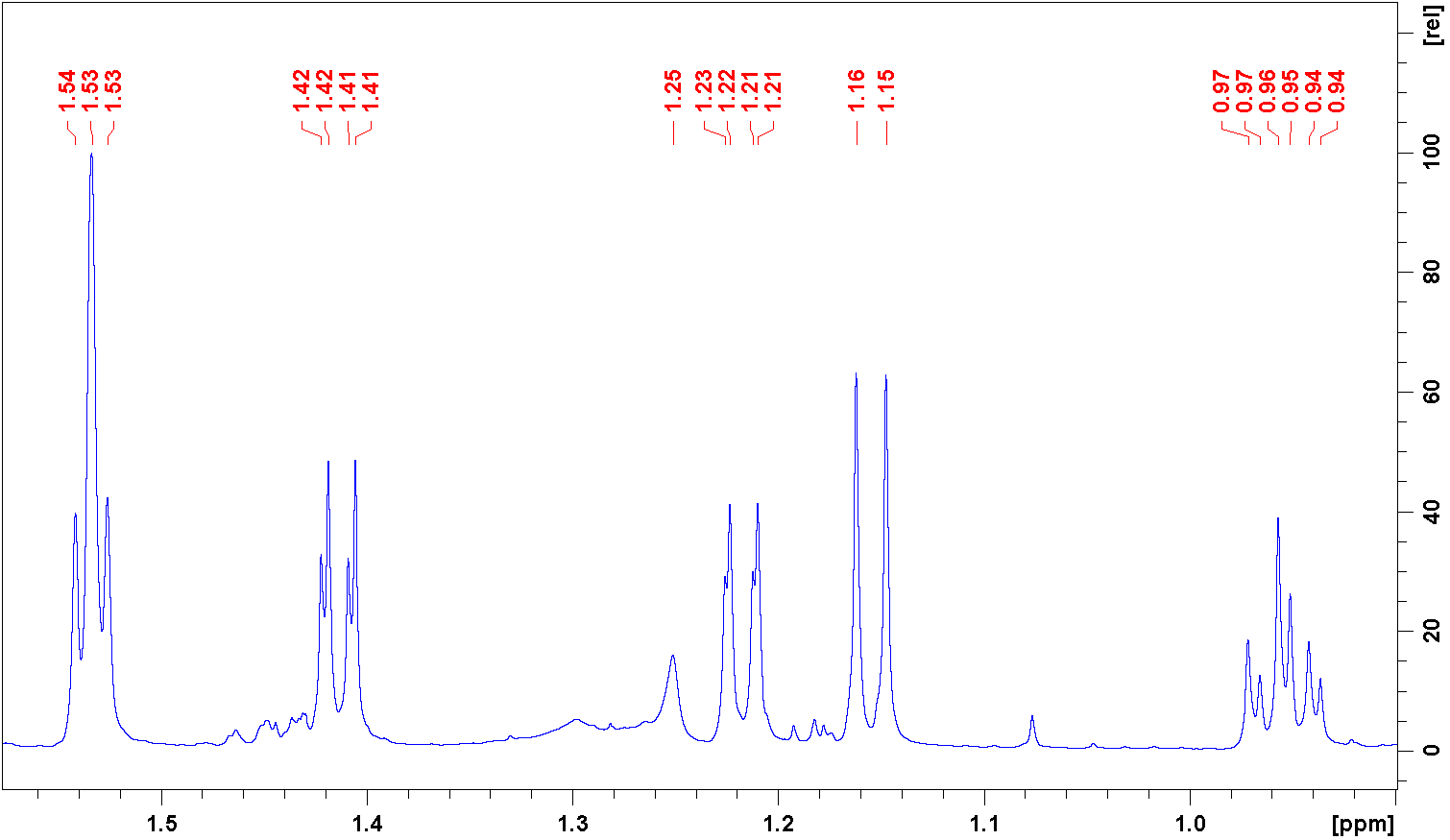

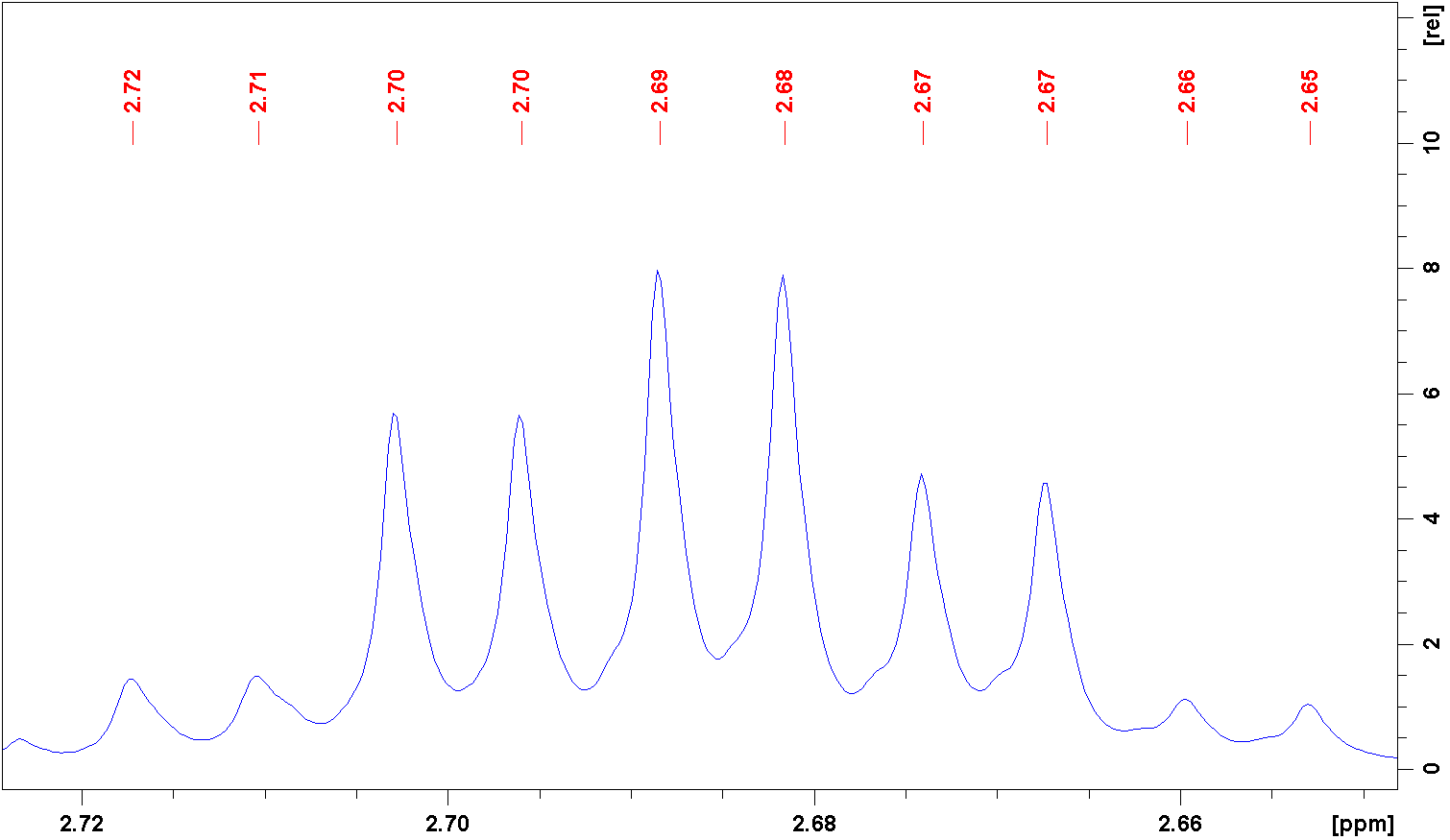

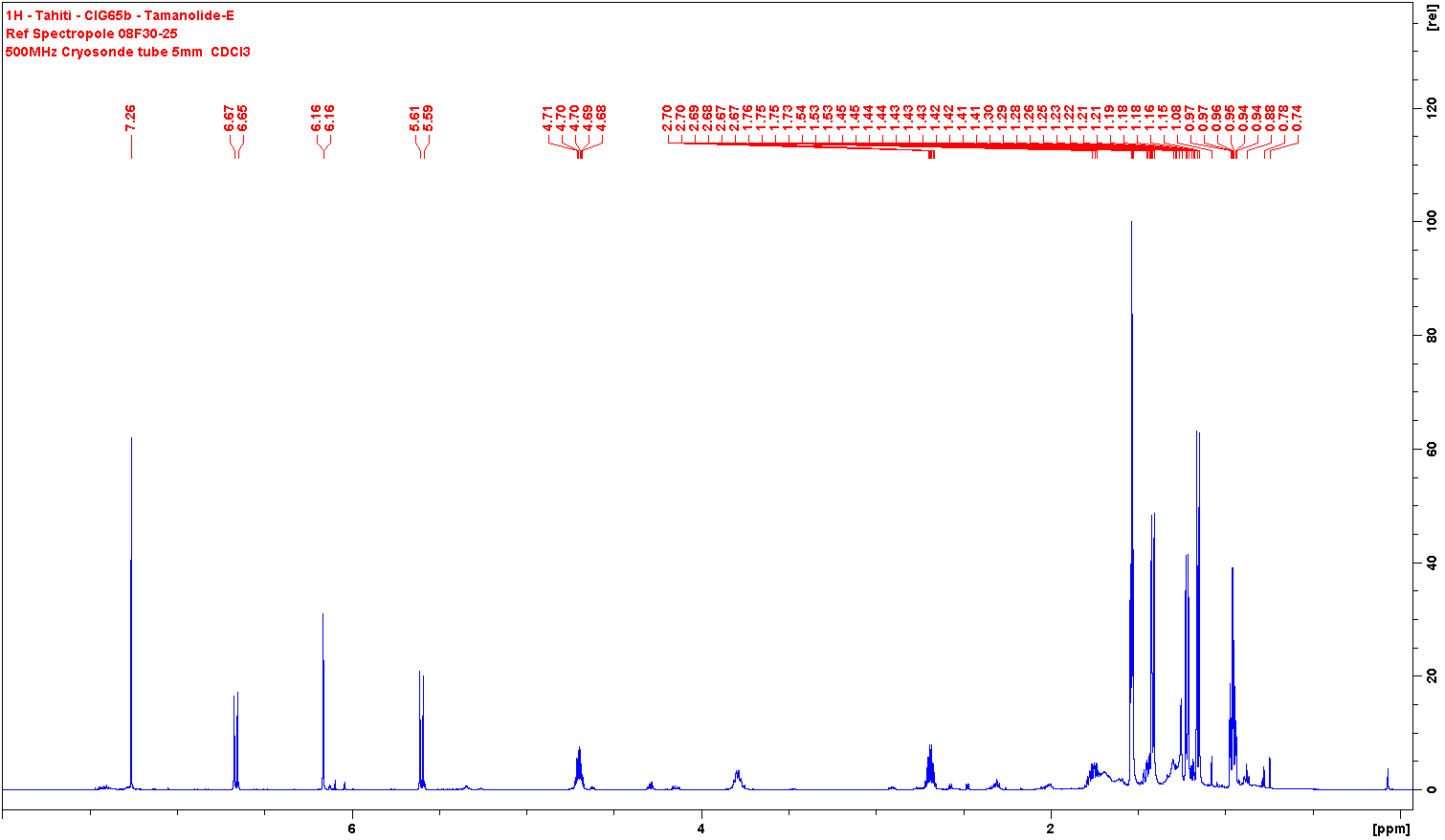


Figure S9 **^1^H NMR Tamanolide E, fraction 2**


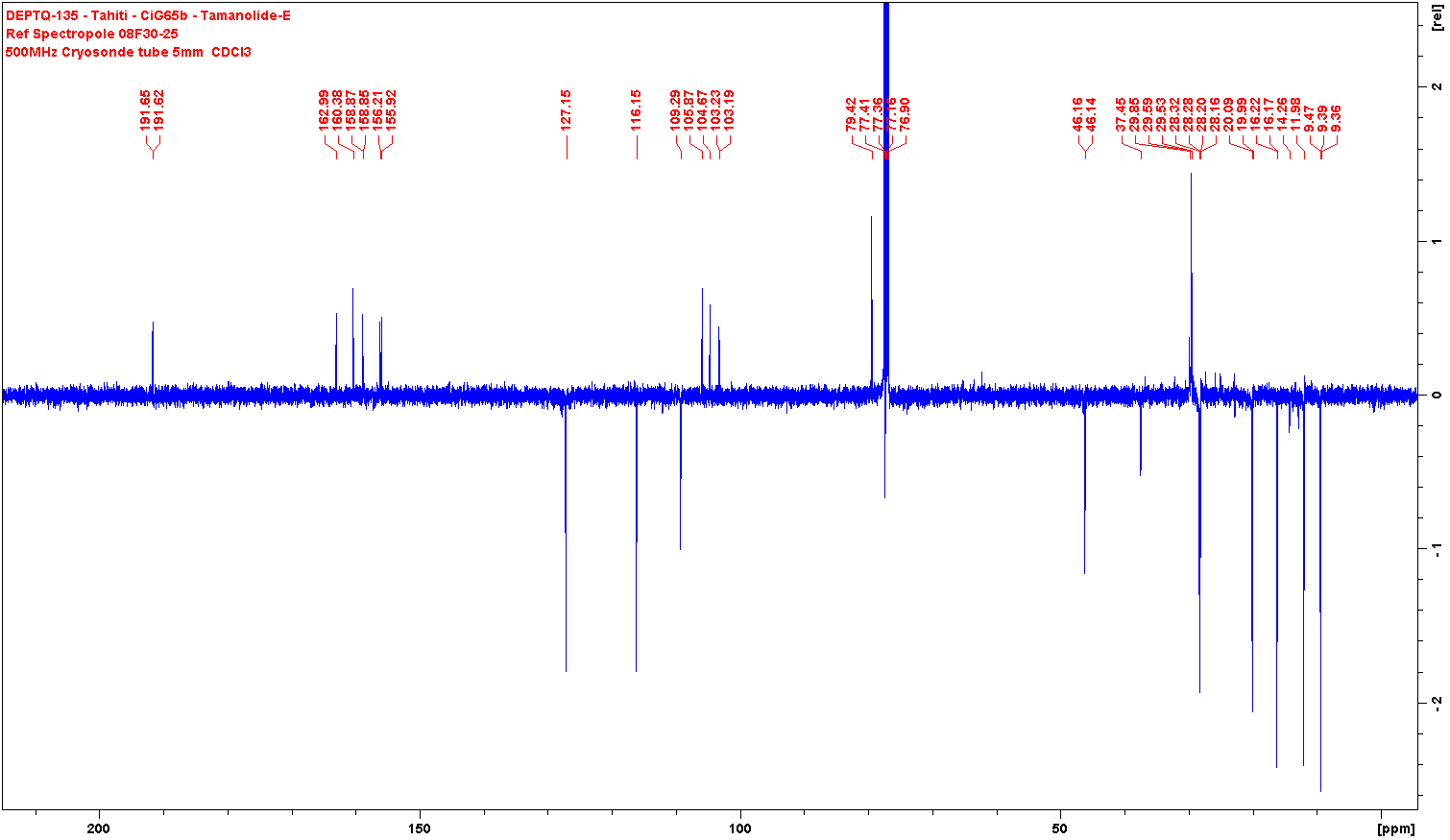


Figure S10 **DEPTQ135 Tamanolide E, fraction 2**
